# Spatio‐temporal precipitation climatology over complex terrain using a censored additive regression model

**DOI:** 10.1002/joc.4913

**Published:** 2016-11-09

**Authors:** Reto Stauffer, Georg J. Mayr, Jakob W. Messner, Nikolaus Umlauf, Achim Zeileis

**Affiliations:** ^1^ Department of Statistics, Faculty of Economics and StatisticsUniversity of InnsbruckAustria; ^2^ Institute of Atmospheric and Cryospheric SciencesUniversity of InnsbruckAustria

**Keywords:** climatology, precipitation, complex terrain, GAMLSS, censoring, daily resolution

## Abstract

Flexible spatio‐temporal models are widely used to create reliable and accurate estimates for precipitation climatologies. Most models are based on square root transformed monthly or annual means, where a normal distribution seems to be appropriate. This assumption becomes invalid on a daily time scale as the observations involve large fractions of zero observations and are limited to non‐negative values.

We develop a novel spatio‐temporal model to estimate the full climatological distribution of precipitation on a daily time scale over complex terrain using a left‐censored normal distribution. The results demonstrate that the new method is able to account for the non‐normal distribution and the large fraction of zero observations. The new climatology provides the full climatological distribution on a very high spatial and temporal resolution, and is competitive with, or even outperforms existing methods, even for arbitrary locations.

## 1 Introduction

Accurate knowledge of precipitation climatology is important for a wide range of applications, such as agriculture, risk assessments, strategic project planning, water resource management, and tourism. Moreover, climatological information is often used as background information for statistical downscaling, or as a baseline for model verification. For locations equipped with a precipitation measurement instrument, this task is straightforward. However, the observational network is generally too sparse to capture all local effects, and observations are preferentially located at lower elevations and close to populated areas due to environmental conditions and maintenance purposes.

To gain information about the amount or occurrence of precipitation for locations without measurements, information from an irregularly spaced observation network has to be brought to a finer (regular) region‐wide grid trough interpolation. Thiessen (1911) pointed out that simple interpolation schemes, such as nearest neighbour, or arithmetic areal means, should not be used for interpolation of precipitation as these methods do not account for local factors that affect precipitation, e.g. distance to mountain ranges, geographical position, and others (Basist *et al*., 1994). Thiessen (1911) invented an areal weighted‐mean scheme that includes terrain‐based properties. Although this was proposed as a first ‘simple’ extension, today's statistical methods still follow a similar idea.

Over recent decades, several different approaches have been developed, which can be clustered into three main classes. The first class consists of ‘exact interpolation schemes’, including inverse distance weighting, and various forms of Kriging (e.g. Biau *et al*., 1999; Goovaerts, 2000). Inverse distance weighting is often not suitable as dependencies on topography, for example, cannot be considered. For Kriging several extensions exist to include additional covariates, or spatio‐temporal Kriging (Snepvangers *et al*., 2003; Aryaputera *et al*., 2015). The second class comprises ‘regional regression models’, where for every location a (simple) regression model is adjusted from only a subset of neighbouring stations. Examples are PRISM (Precipitation‐elevation Regressions on Independent Slopes Model; Daly *et al*., 1994, 1997, 2002, 2008), and Daymet (Thornton *et al*., 1997).

A third class of interpolation methods consists of ‘smooth spline regression models’, which are the focus of this article. Generalized additive models (GAMs; Guisan *et al*., 2002) are a common form of smooth spline models, where a response quantity is described by a set of possibly nonlinear functions of covariates. Feasible functions could be an altitudinal effect, a cyclic effect to represent the seasonality, or a two‐dimensional spline on longitude and latitude to describe the spatial distribution. Spline models have already been used for long‐term climatologies for different quantities, such as temperature or precipitation (e.g. Boer *et al*., 2001; Jarvis and Stuart, 2001; Vicente‐Serrano *et al.*, 2003; Guan *et al*., 2009).

However, regarding precipitation, most studies have focused only on monthly or even annual means.

Finer than monthly temporal resolution is needed for a wide range of applications, so climatologies limited to monthly sums are unsatisfying. Furthermore, additional useful properties of the climatological distribution are of great interest, such as the probability of precipitation, or specific quantiles. This can be achieved by either creating a specific statistical model for each of the quantity of interest, or by modelling the full climatological distribution in one model. Such fully distributional climatological estimates can be used for statistical downscaling approaches based on, for example, quantile mapping or model output statistics. Quantile mapping is often used to calibrate climate or weather forecast models by re‐sampling the forecasted distribution from the observed distribution (Themeßl *et al*., 2012; Acharya *et al*., 2013; Ajaaj *et al*., 2016; Rajczak *et al*., 2016). Climatological estimates are also used as background information for spatial model output statistics methods to account for site‐specific climatological features not yet resolved by the numerical weather or climate models (Scheuerer and Büermann, 2014; Dabernig *et al*., 2016; Stauffer *et al*., 2016). Furthermore, climatological estimates are useful as baseline verification. Fully probabilistic spatial climatologies provide all necessary information to compute a wide range of verification scores, such as Brier scores, root mean squared errors (RMSE), but also probabilistic scores like the continuous rank probability score (CRPS), or quantile score.

**Figure 1:**
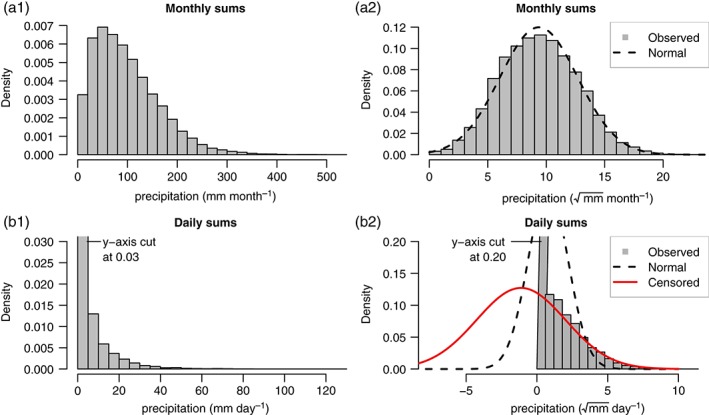
Density plot of precipitation sums. Top row: monthly precipitation sums from all 117 stations. Bottom row: daily precipitation sums of one sample station (‘Iselsberg‐Penzelberg’). Right column: power‐transformed observations ($\sqrt{\mathrm{mm}}\;{\text{day}}^{-1}$) with a fitted normal distribution (black, dashed). In addition, a fitted zero left‐censored normal distribution is shown for the daily power‐transformed observations (b2; red, solid). Please note: y‐axes in the bottom row are both cut. [Colour figure can be viewed at wileyonlinelibrary.com].

An accurate estimate of the full climatological distribution requires a suitable response distribution. Figure 1(a1) shows an example of monthly precipitation sums of 117 stations, which are strongly skewed to the right (positive skewness). To remove the skewness, a power transformation has been used frequently (Box and Cox, 1964). In literature, cubic (Stidd, 1973) or square root (Hutchinson, 1998a) transformations have often been suggested but may vary for different climatic zones or temporal aggregation periods. After applying a square root transformation (Figure 1(a2)) the majority of the skewness is removed and the data are close to a normal distribution (dashed line). Aggregation into monthly sums usually moves the data away from zero yielding a pseudounbounded data set (Sansom and Tait, 2004), wherefore the assumption of a normal distribution might be appropriate. However, daily precipitation sums show different properties. Figure 1(b1) shows all daily sums of station ‘Iselsberg‐Penzelberg’ (later referred to as station B). Three main properties can be identified:

- The distribution is strongly positively skewed,
- the distribution is limited to non‐negative values (≥0), and
- a large fraction of all observations is exactly zero (dry days).

To remove the characteristic skewness a power transformation can be applied, the remaining properties must be accounted for separately by assuming a proper response distribution. For hourly or daily precipitation sums that remain physically limited to non‐negative values after the power transformation (Figure 1(b2)). Several studies have shown that the concept of censoring works well for precipitation, as precipitation is physically limited to non‐negative values (Messner *et al*., 2014; Scheuerer, 2014; Scheuerer and Hamill, 2015).

In this article, we present a novel spatio‐temporal additive model with a zero left‐censored normal response, to estimate a full‐distributional climatology of precipitation over complex terrain on a daily temporal resolution. To obtain both, the climatological mean and the climatological variance, a distributional regression model (Klein *et al*., 2015) is used. Distribution regression allows all parameters of a response distribution to be modelled by a set of explanatory covariates. Statistical frameworks that allow for distributional regression are often termed as vector generalized additive models (VGAM; Yee, 2015) or generalized additive model for location, scale, and shape (GAMLSS; Rigby and Stasinopoulos, 2005). We use a GAMLSS model to obtain the climatological estimates for each day of the year, and for any arbitrary location within the study area. A power transformation is used to deal with the skewness, while assuming a zero left‐censored normal distribution handles both the lower limit at zero, and the large fraction of zero observations in the data set. This new approach allows full scalability (size of the area of interest, but also spatial‐ and temporal resolution) and can therefore be implemented easily and applied to new data sets or regions.

## 2 Methodology

### 2.1 Left‐censored normal distribution

The response distribution of the model is crucial to its overall performance. In contrast to the observed monthly sums, even after the power transform, daily values show a strong peak at zero caused by the large fraction zero observations (days without precipitation). The concept of censoring is that the response itself is limited to a certain threshold *τ*, or a range, and cannot be observed outside these limits. It is assumed that there is a latent unobservable process driving the response, which can be described by suitable covariates. As precipitation is physically limited to 0 mm, it can be seen as left‐censored at zero (*τ* = 0). The resulting zero left‐censored normal distribution is specified as follows:

(1) $$y=\max\;\left(0,,,y^{*}\right),\;y^{*}\;∼N\left(\mu ,,,\sigma \right)$$

y* denotes the unobservable ‘latent’ response following a normal distribution, given the parameters location *μ* and scale *σ*. The ‘observable’ response *y* is simply the maximum of the latent response and the censoring point. From here on this distribution will be denoted as *N*_0_. The density (*ϕ*_cens_) and the distribution function (*Φ*_cens_) for *N*_0_ can be written as follows:

(2) $$\phi_{\text{cens}}\left(x_{i}|\mu ,\sigma ,0\right)=\left\{\begin{array}{cc} 0 & \;for\;x_{i}<0\\ \Phi \left(x_{i}|\mu ,\sigma \right) & \;for\;x_{i}=0\\ \phi \left(x_{i}|\mu ,\sigma \right) & \;\text{else} \end{array}\right.$$

(3) $$\Phi_{\text{cens}}\left(x_{i}|\mu ,\sigma ,0\right)=\left\{\begin{array}{cc} 0 & \;for\;\text{all}:\;x_{i}<0\\ \Phi \left(x_{i}|\mu ,\sigma \right) & \;\text{else} \end{array}\right.$$

While both quantities are set to zero below the censoring point, both follow the density *Φ* and distribution function *Φ* of a non‐censored normal distribution, respectively, above the censoring point (*x*_*i*_ > 0). On the censoring point (*x*_*i*_ = 0), the distribution function is again equivalent to the normal distribution, while the density represents the probability that an observation will lie exactly on zero. Therefore, the probability *π* to exceed zero can be written as:

(4) $$\pi \left(y>0\right)=1-\Phi \left(0|\mu ,\sigma \right)$$

A last property of interest is the expectation of *N*_0_. As the estimates will be fitted on a power‐transformed scale *y* with *y* = *z* ^1/*p*^, this transformation has to be included to get the expectation on the original scale (mm day^−1^). The expectation function of a power‐transformed *N*_0_ can be expressed as (Appendix):

(5) $$E\left[z\right]=\int_{0}^{\infty }z\cdot \phi \left(z^{\frac{1}{p}}|\mu ,\sigma \right)\cdot \frac{z^{\left(\frac{1}{p}-1\right)}}{p}dz$$

where *z* is the observable response on the original scale, *μ* and *σ* are the estimated parameters of *N*_0_ on the power‐transformed scale, and *p* denotes the power parameter of the power transformation.

Figure 1(b2) shows the fitted zero left‐censored normal distribution (solid line) which is able to depict the distribution of the data very accurately. Comparing the estimated (44%) and observed (43%) probability of precipitation, as well as the estimated (2.3 mm day^−1^) and observed (2.2 mm day^−1^) expectation shows that the power‐transformed zero left‐censored normal distribution is able to account for the large fraction of zero observations, and to accurately adjust the distribution of the non‐censored part.

### 2.2 Generalized additive models for location, shape, and scale

GAMLSS (Rigby and Stasinopoulos, 2005) are an extension to GAMs (Guisan *et al.*, 2002) that allow all parameters of a certain response distribution to be modelled separately. In case of a censored normal distribution two parameters have to be specified: ‘latent’ location (mean), and ‘latent’ scale (standard deviation). For a zero left‐censored normal distribution (*N*_0_) the GAMLSS model can be expressed as follows:

(6) $$\begin{array}{c} y∼N_{0}\left(\mu ,,,\sigma \right)\\ \mu =s\left(x\right)\\ \text{log}\left(\sigma \right)=t\left(x\right) \end{array}$$

The ‘observable’ response *y* is assumed to follow *N*_0_ with location *μ* and scale *σ*, where the log‐link ensures positive scale values during optimisation. Both parameters can be expressed by a set of unknown, possibly nonlinear functions *s*(…) and *t*(…), also known as linear predictors. The explanatory variables **x** include the covariates, such as altitude, longitude, latitude, or others.

In the GAMLSS framework, the linear predictors can include different additive effects, such as linear effects, nonlinear effects, cyclic effects, or two‐dimensional surfaces. For nonlinear one‐dimensional and multi‐dimensional effects, splines are used very frequently. Common forms of splines are, e.g. thin‐plate splines, or B‐splines (Wood, 2006, Chap. 4.1, Fahrmeir *et al*., 2013). As complex splines tend to get wiggly, an additional penalization term is estimated yielding to smooth regression splines.

For applications where only the mean is of interest, the scale parameter in Equation (6) could be specified as a constant. The result would be a homoscedastic GAM model where the variance is constant among all observations. Models of this type have been used frequently for the application of precipitation climatologies, such as in Hutchinson (1998a, 1998b); Price *et al.* (2000); Boer *et al.* (2001); Hong *et al.* (2005). However, as we would like to estimate the full daily climatological distribution, the linear predictor for *log*(*σ*) in Equation (6) has to be specified in addition.

For the specific application of a spatio‐temporal precipitation climatology, the effects *s*(**x**) and *t*(**x**) have to capture a possible altitudinal effect, the seasonality, as well as the spatial pattern. Therefore, the following effects have been specified for parameter *μ* (location):

(7) $$\begin{array}{c} \mu =s\left(x\right)=\beta +s_{1}\left(\text{alt}\right)+s_{2}\left(\text{yday}\right)+\\ s_{3}\left(lon,\;lat\right)+s_{4}\left(\text{yday},\;lon,\;lat\right) \end{array}$$

where *β* denotes the global intercept, *s*_1_(alt) represents a smooth ‘altitudinal’ effect, *s*_2_(*y*day) a cyclic seasonal effect based on the ‘day of the year’, *s*_3_(lon, lat) a two‐dimensional spatial effect given the geographical coordinates ‘longitude’ and ‘latitude’, and *s*_4_(yday, lon, lat) represents a three‐dimensional spline to account for spatial variabilities of the seasonal pattern across the region of interest. Cubic splines are used for the seasonal effect as they allow addition of a cyclic constraint such that no discontinuities emerge between December and January. All other effects use thin‐plate regression splines. Thin‐plate regression splines use the Eigenbasis of the data and ‘provide optimal low rank approximations to thin‐plate splines that are both computationally efficient and stable’ (Wood, 2003; p. 110) typically leading to better results.

Analogously to the linear predictor for the location *μ* (Equation (7)), the linear predictor for the log‐scale is expressed as follows:

(8) $$\begin{array}{c} \text{log}\left(\sigma \right)=t\left(z\right)=\gamma +t_{1}\left(\text{alt}\right)+t_{2}\left(\text{yday}\right)+\\ t_{3}\left(lon,\;lat\right)+t_{4}\left(\text{yday},\;lon,\;lat\right) \end{array}$$

The two linear predictors include the same effects, as we expect the climatological variance for precipitation to also show a seasonal and spatial dependency, as well as an altitudinal effect (Equations (7) and (8)). This seems appropriate for the specific task of this article, but is no general requirement for GAMLSS models.

### 2.3 Model setup

To estimate the nonparametric smooth model as specified in Equations (6)–(8) suitable software is required which allows for a zero left‐censored normal distribution. We are using a novel *R* package ‘bamlss’ (Umlauf *et al*., 2016b) which offers a flexible Bayesian framework for additive models for location, scale, and shape (and beyond), and the capability to handle (very) large data sets. Other possible software implementations to estimate smooth models are, e.g. ANUSPLIN (Hutchinson, 2014), or the R packages ‘mgcv’ (Wood, 2006) and ‘gamlss’ (Rigby and Stasinopoulos, 2005).

In addition to the full spatio‐temporal climatology station‐wise models are estimated for comparison. These station‐wise models use the same technique, but as only the information of one station is used, the spatial effects are not required. The station‐wise models therefore only include the intercepts (*β*, *γ*) and the seasonal effects [*s*_2_(yday), *t*_2_(yday)], while all other assumptions are equivalent to Equations (6)–(8).

The skewness is removed by applying a power transformation to the observations. In previous studies, the empirical power parameters *p* = 2 (square) or *p* = 3 (cubic) have been used. However, the power parameter depends on the data and the response distribution of the application. To obtain the best power parameter for this study, we fitted one station‐wise GAMLSS model for each station in the data set, optimizing the regression coefficients plus an additional power parameter simultaneously. Optimal power parameter did not show an obvious spatial or altitudinal dependency and varied between *p* = 1.3 and *p* = 2.0. Tests have shown that the model performance is not very sensitive within this range, therefore, a fixed value of *p* = 1.6 corresponding to the median of all estimated power coefficients was chosen.

The estimates for all GAMLSS models (‘station‐wise’ and ‘spatio‐temporal’; Section 4) are based on the new *R* package bamlss. The optimisation is based on Markov‐Chain Monte Carlo (MCMC) sampling in combination with an iterative weighted least squares backfitting algorithm (Umlauf *et al*., 2016a). Code and data used in this article can be downloaded from the bamlss project page (http://bayesr.r‐forge.r‐project.org/).

## 3 Area of interest and data

**Figure 2:**
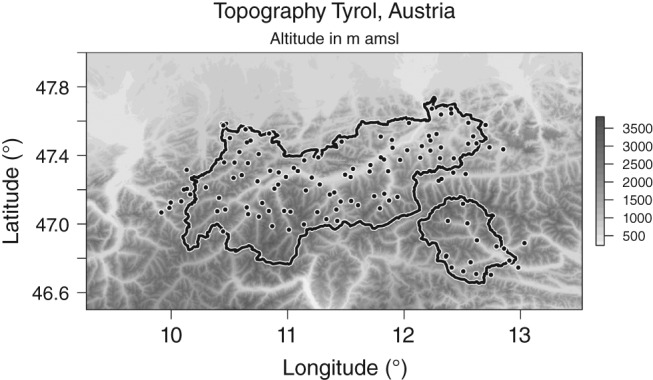
Topography around Tyrol, Austria. Shading indicates altitude of the topography in metres, the outline shows the border of the state of Tyrol consisting of North and East Tyrol. The black dots show the stations locations from the data set.

This article focuses on the temperate alpine state of Tyrol, Austria, located in Central Europe. Tyrol lies in the Eastern Alps and consists of two separated parts – North Tyrol located north of the main Alpine ridge, and East Tyrol located south of the main Alpine ridge, as shown in Figure 2. The topography reaches from 465 up to 3798 m amsl including the majority of the highest mountains in Austria. This complexity is one of the main difficulties from a climatological perspective, as climatological properties can strongly vary within just a few kilometres due to topographically induced effects.

Compared to other regions, Tyrol has a relatively dense precipitation observation network with a mean station distance of about 10 km. The observation data set used in this study is provided by the local hydrographical service and includes 117 stations (Figure 2) spanning September 1971 through the end of 2012. A total of 78 out of 117 stations include at least 40 years of data, 14 start within the 1980s, nine within the 1990s and three post‐millennial. Each station is equipped with a manual rain gauge to measure liquid water or liquid water equivalent accumulated over the last 24 h, observed at 0600 UTC. The hydrographical service performs rigorous quality controls on the observations. The total data availability is about 88% equating to ca 1.6 million unique daily observations. For a region with such a complex topography, a dense observational network is essential to be able to depict all small‐scale spatial features, and also the pronounced altitudinal effects. For regions with less complex topographic features, a sparser network might be sufficient as the spatial differences within the area will presumably be smaller. The data set used in this study is freely available for non‐commercial use, and can be downloaded from the bamlss project page (BMLFUW; http://bayesr.r‐forge.r‐project.org/).

**Figure 3:**
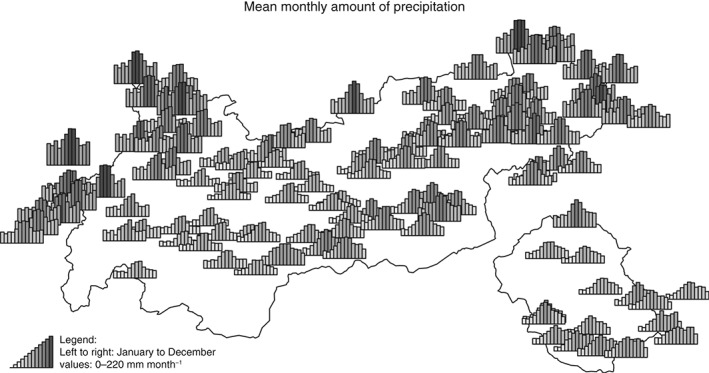
Mean monthly precipitation based on the data set. Each bar indicates 1 month (January–December, left to right). Bar height and luminance contain the same information (0–220 mm month^−1^).

Figure 3 shows the mean monthly precipitation sums for all stations. The largest amounts of precipitation with around 1100–2100 mm per year are observed for the north‐west and north‐east stations, and a second slightly weaker maximum with >1000 mm per year for the south‐east stations. This is due to the proximity to the foreland of the Alps (Bavaria, Germany to the north, northern Italy to the south) and dynamically driven processes. Incoming air masses are lifted when they encounter the first obstacles, leading to orographic precipitation, and a loss of moisture at the foot of the Alps (Houze, 2012). On the north side, this effect is mainly caused by fronts advected from north‐westerly directions, leading to higher mean precipitation amounts over the whole year. In the south‐east, the highest precipitation amounts are related to mesoscale cyclones forming over the Mediterranean sea (e.g. Raulin, 1879; Frei and Schär, 1998). All stations show a local maximum in summer (June–August), which is mainly caused by local thermal convection, which leads to increased amounts of precipitation and thunderstorms. The convective enhancement is strongest in the pre‐alpine regions north‐west, and north‐east of Tyrol (Wapler, 2013).

## 4 Results

First, the estimated effects of the new censored spatio‐temporal precipitation climatology will be shown in Section 4.1, followed by a model comparison and validation.

### 4.1 Results of the new daily‐based spatio‐temporal model

**Figure 4:**
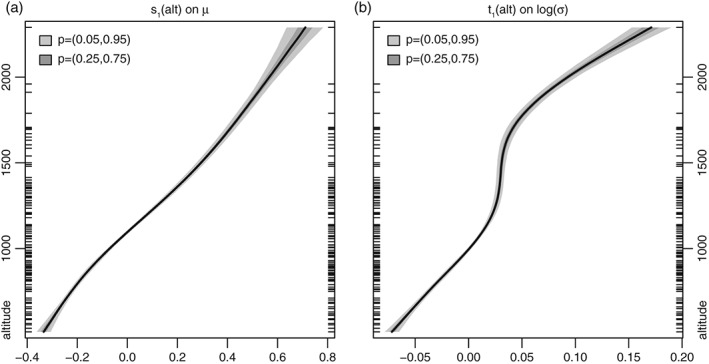
Centred altitudinal effects s_1_(yday) on location μ (a), and t_1_(yday) on log(σ) (b). Values on the power‐transformed scale. Inner ticks on the ordinate indicate the altitudes of all stations in the data set. The shading shows the confidence intervals of the estimate and the width is closely related to the large amount of training data.

**Figure 5:**
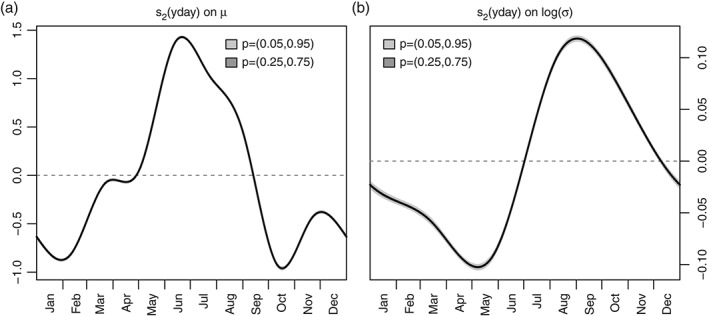
Centred cyclic seasonal effects s_2_(yday) on location μ (a), and t_2_(yday) on log(σ) (b). Values on the power‐transformed scale. The shading shows the confidence intervals of the estimate, the width is closely related to the large amount of training data. The effect controls the global seasonal effect for all stations.

**Figure 6:**
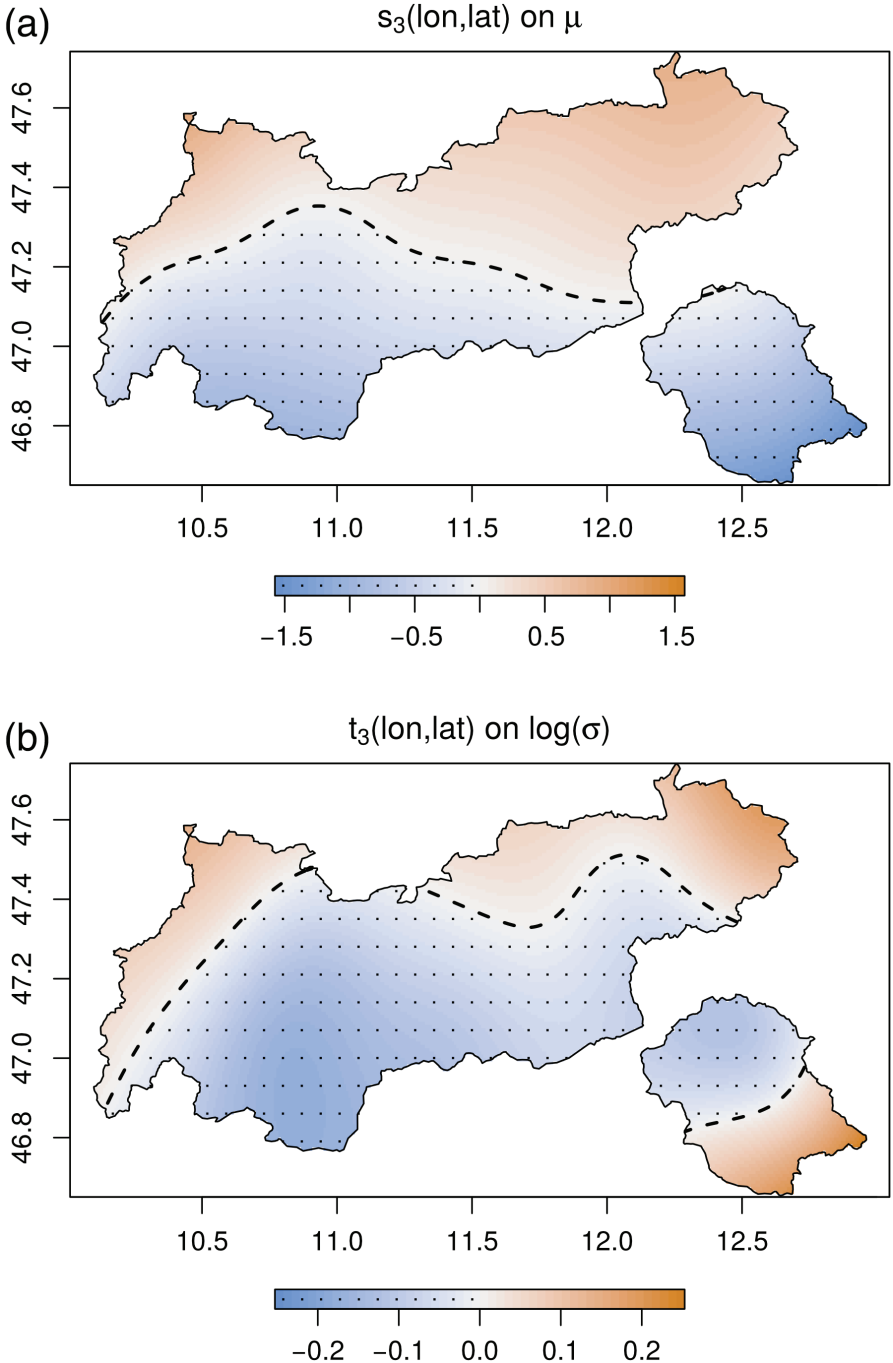
Centred spatial effect s_2_(lon , lat) on location μ (a), and t_3_(lon , lat) on log(σ) (b). Values on the power‐transformed scale. Positive values orange, negative values blue and additionally dotted. The effect controls the mean underlying climatological spatial distribution of precipitation. [Colour figure can be viewed at wileyonlinelibrary.com].

**Figure 7:**
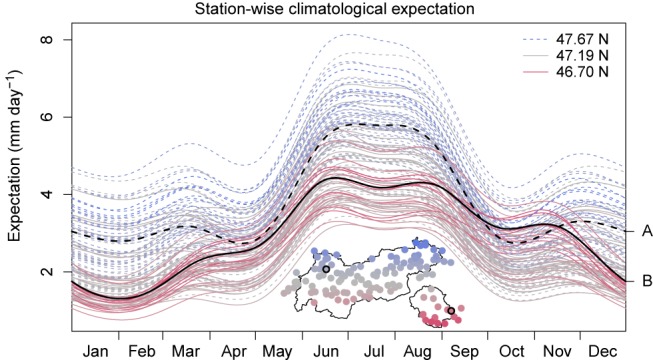
Expectation in mm day^−1^ for all 117 stations used. Stations are coded blue/dashed to the north, and red/solid to the south. The two sample stations A and B as shown in Figure 8 are highlighted in black. The difference in the seasonal pattern between north and south results from the tri‐variate thin‐plate splines s_4_, t_4_ based on the day of the year, longitude, and latitude. [Colour figure can be viewed at wileyonlinelibrary.com].

As described in Section 2.3 a spatio‐temporal GAMLSS with a zero left‐censored normal response is used to create the long‐term precipitation climatologies (Equation (6)) with the linear predictors for location (*μ*) and log‐scale (*log*(*σ*)) as specified in Equations (7) and (8). The individual effects of the two linear predictors are shown in Figures 4, 5, 6, 7. All figures, except the last, show centred effects on the power‐transformed scale.

Figure 4 shows the altitudinal effects for location *μ* (left), and log‐scale (right). As expected, the amount of precipitation and the variance increase with increasing altitude (Ekhart, 1948; Frei and Schär, 1998). The global cyclic seasonal effects for location *μ* (left) and log‐scale (right) are shown in Figure 5. The seasonal effect shows the overall dry winter conditions from December to February (compare Figure 3) with a low variability. Overall, June–August are the months showing largest amounts of precipitation, with increasing variability during mid to late summer related to the convective season with its peak between July and September. During this time period, location *μ* already decreases, while the log‐scale nearly reaches its overall maximum. Or in other words: in autumn, the overall amount of precipitation strongly decreases (relatively dry), but the variability reaches its local annual maximum. October is the driest month but still shows high variability compared to the first half of the year.

The spatial effects are shown in Figure 6. As for the seasonal cycle, location *μ* and log‐scale show different patterns. While location *μ* increases from south to north, the log‐scale effect reaches its maximum towards the pre‐alpine plains with Bavaria, Germany to the north, and Italy to the south. The increase in location *μ* is related to fronts reaching Tyrol predominantly from north and north‐westerly directions. The increase in the variability is mainly caused by higher convective activity (Wapler, 2013), and the orographic precipitation produced when air masses approaching from plains encounter the first higher obstacles.

Seasonal patterns differ for different regions. The three‐dimensional thin‐plate splines *s*_4_, *t*_4_ in Equations (7) and (8) allow for a spatial variation of the cyclic seasonal pattern across the area of interest. This effect can be seen in Figure 7, which shows the estimated climatological expectation in mm day^−1^ for all 117 stations in the data set. The results show that the new climatology is able to capture the different seasonal characteristics between the sub‐regions north and south of the main Alpine ridge.

**Figure 8:**
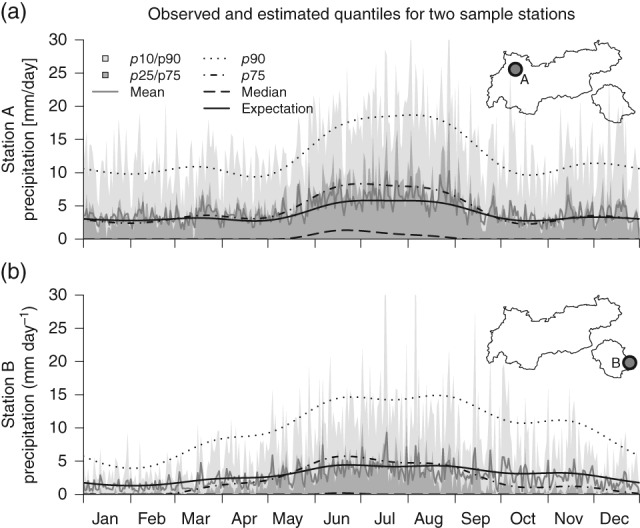
Distribution of daily observed precipitation sums for two sample stations. The long‐term daily distribution is shown in grey including 42 years of observations: 10–90% and 25–75% inner‐quantile ranges (shaded), and mean (solid, grey). In addition, the climatological estimate from the spatio‐temporal model is shown. Expectation (Equation (5)) as solid, and quantiles as black lines of different styles. Mean annual precipitation sums/frequency of observed precipitation for both stations: station A ‘Namlos’ (a) 1577 mm year^−1^/48%, station B ‘Iselsberg‐Penzelberg’ (b) 954 mm year^−1^/36%. Table 1 contains CP for both stations.

As the new climatology returns estimates for the full distribution, it is also possible to examine other properties, such as quantiles or the probability of precipitation: Figure 8 shows the climatological distribution and the corresponding climatological estimates for two sample stations of the data set. Station A is located north of the main Alpine ridge and close to the pre‐alpine foreland. Station B lies south of the main Alpine ridge. A few distinct features can be identified. Station A receives precipitation more frequently and observes larger amounts of precipitation than station B. Furthermore, the different seasonality can be seen. While station A shows a clear summer‐signal with a strong increase during May–June and a corresponding decrease in autumn, station B shows a smoother transition across the year, with an overall lower amplitude. The censored daily spatio‐temporal climatology captures the main features of amplitude, seasonality, and the overall distribution.

In addition, Table 1 contains coverage probabilities (CP) of the spatio‐temporal estimates for the two sample stations in Figure 8. The CP shows the fraction of observations falling into the verification interval and therefore shows a measure of calibration. For the two intervals [0.10–0.90] and [0.25–0.75], the theoretical or perfect coverage would be 0.80 and 0.50, respectively. The validation shows 0.78/0.46 for station A and 0.82/0.51 for station B indicating that the estimates are a bit overdispersive (too wide) for station A, and slightly underdispersive (too narrow) for station B.

**Table 1:** CP for station A and station B (Figure 8), and for all 117 stations (overall) for the intervals [0.10–0.90], [0.25–0.75], and [0.00–0.50]. Sample CPs for the spatio‐temporal GAMLSS (top) and the station‐wise GAMLSS (bottom). Theoretical CPs are shown in parenthesis.

Interval	Station A	Station B	Overall
*Spatio‐temporal GAMLSS*			
0.10–0.90 (0.80)	0.78	0.82	0.81
0.25–0.75 (0.50)	0.46	0.51	0.50
0.00–0.50 (0.50)	0.49	0.51	0.49
*Station‐wise GAMLSS*			
0.10–0.90 (0.80)	0.80	0.81	0.81
0.25–0.75 (0.50)	0.49	0.50	0.50
0.00–0.50 (0.50)	0.49	0.49	0.49

**Figure 9:**
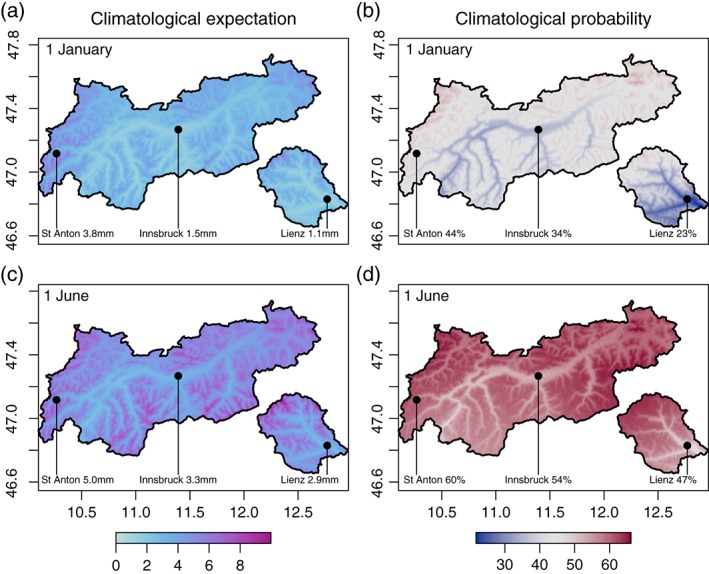
Climatological expectation (a and c; mm day^−1^), and climatological probability of precipitation (b and d; %) for 1 January (a and b), and 1 June (c and d), respectively. Values are explicitly shown for three locations: St Anton (1284 m amsl), Innsbruck (574 m amsl), and Lienz (673 m amsl). Prediction based on the SRTM DEM (CGIAR‐CSI, 2016).

Figure 9 shows the spatio‐temporal climatology for two sample days, 1 January (top), and the 1 June (bottom). The climatological expectation (left column) shows the overall drier winter conditions and the distinct altitudinal dependence with up to ∼7 mm day^−1^ on 1 January, and up to ∼10 mm day^−1^ on 1 June. The right column shows the probability of precipitation in percent. On 1 January, the highest probability of observing precipitation is towards the foreland to the north, while the inner‐alpine regions close to the main Alpine ridge show relatively low probabilities. On 1 June, the overall probability of precipitation increases, with probabilities above ∼55% for all mountainous areas.

### 4.2 Model comparison and validation

The novel spatio‐temporal precipitation climatology validated in consideration of a number of aspects of model performance. Of special interest is the performance for fully out‐of‐sample events to show the predictive performance for future (temporally out‐of‐sample) events at arbitrary locations within the area of interest (spatially out‐of‐sample). Consequently, the out‐of‐sample predictions of the spatio‐temporal model will be compared against two in‐sample station‐wise reference methods. All three models are trained on observations through the end of 2009, including up to 39 years of data (Section 3), evaluated on the remaining 3 years between 2010 and 2012, from here on referred to as ‘training’ and ‘test data set’.

#### 4.2.1 Monthly mean model

As a robust and simple baseline reference model, long‐term monthly means of the measurements are computed for each station separately. Similarly, the probability of precipitation is the long‐term mean frequency of observations greater than zero for a given station and month. Months with missing data are excluded.

#### 4.2.2 Station‐wise GAMLSS

To validate the goodness of fit of the spatial effects of the spatio‐temporal model, station‐wise GAMLSS climatologies with a zero left‐censored normal distribution have been estimated. One model is estimated for each of the 117 stations using Equations (6)–(8) with modified linear predictors. As these models are station‐wise, only the intercepts and seasonal effects have to be included.

#### 4.2.3 Spatio‐temporal GAMLSS

To score the predictive skill of the novel spatio‐temporal climatology, a ten‐fold cross‐validation is performed. For each cross‐fold, a random subset including 10% of all stations is omitted. The spatio‐temporal model is estimated on the remaining stations using the specifications of Equations (6)–(8). For the left‐out 10% of the stations, the predictions are made on the test data set. This leads to ‘spatially out‐of‐sample’ predictions, while both station‐wise methods are ‘spatially in‐sample’.

#### 4.2.4 Measure of performance

**Figure 10:**
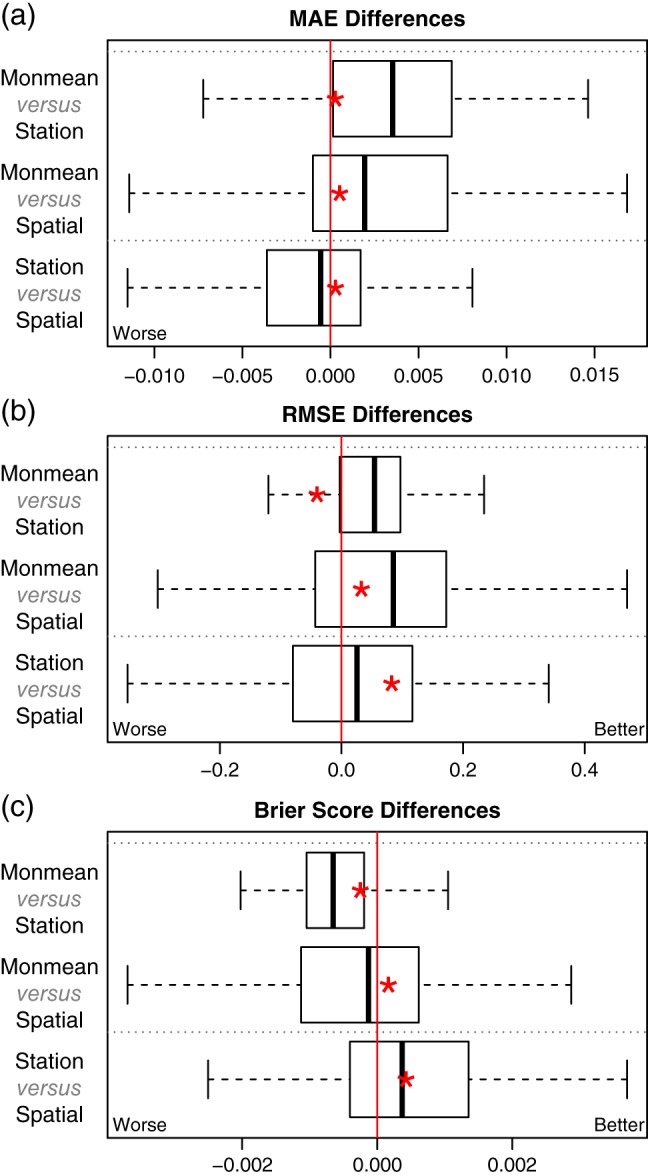
Differences in MAE, RMSE, and Brier scores for all model pairs: monthly mean model (monmean), station‐wise GAMLSS (station), and spatio‐temporal GAMLSS (spatial). Each box‐whisker consists of 117 station‐wise values, each of which is the mean error for one specific station. Box‐whiskers show the results on the test data set (0.25/0.5/0.75 quantiles plus additional 1.5 inner‐quantile range) and the red asterisk indicates the median of the same analysis on the training data set. The differences are defined as ‘method B − method A’ such that positive values indicate that ‘method A’ performs better than ‘method B’ for each ‘A versus B’. Absolute values lie around 3.35 (MAE), 7.25 (RMSE), and 0.24 (Brier score). [Colour figure can be viewed at wileyonlinelibrary.com].

As a measure of performance, mean absolute errors (MAEs), RMSE, and Brier scores (Brier, 1950) will be shown. While the first two are used for the amount of precipitation, the Brier scores show the performance on the estimated probability of precipitation. MAEs are based on the median of the climatological distribution (max(0, *y*)*^p^*; Equation (1)), while the RMSE are based on the expectation (Equation (5)). The Brier scores depend on the probability that precipitation will be observed (Equation (4)). A Brier score of zero would indicate a perfect forecast. To compare the different models, error‐differences are shown in Figure 10. Each box‐whisker is based on 117 values, each of which is the mean error difference of a specific station. The error‐differences are shown between each pair of methods, where the difference is defined as ‘method B − method A’ threading ‘method B’ as the reference. For example: Figure 10(a) shows the differences in the MAE, where the first pair shows ‘monthly mean model (monmean) *versus* station‐wise GAMLSS (station)’. On the test data set the ‘monthly mean model’ performs slightly better than the ‘station‐wise GAMLSS’, while both are more or less identical (in median) evaluated on the training data set. Figure 10(b) and (c) shows the same validation for the RMSE, and Brier score respectively. The novel spatio‐temporal zero left‐censored GAMLSS model shows comparable results in all measures, or indeed slightly better in terms of Brier scores, even if the predictions of the spatio‐temporal GAMLSS model are the only ones which are fully out‐of‐sample.

**Figure 11:**
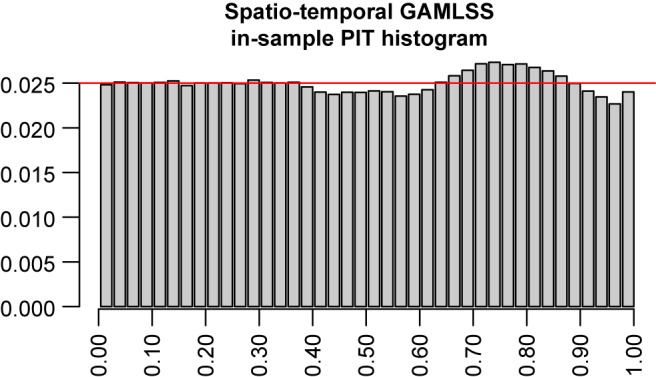
Pit histogram of the spatio‐temporal GAMLSS model evaluated on the training data set. Width of the bins: 2.5%. [Colour figure can be viewed at wileyonlinelibrary.com].

A probability integral transform (PIT; Gneiting *et al*., 2007) histogram is shown in Figure 11 to check the suitability of the fitted climatological distributions. The PIT histogram contains CP for a set of evenly distributed non‐overlapping intervals. For each observation, the corresponding quantile of the climatological distribution is evaluated and then pooled into bins. A perfectly calibrated model would show a uniform distribution across all bins. The PIT histogram indicates that the zero left‐censored normal distribution seems suitable for the application of precipitation, but the deviation from a perfectly uniform distribution indicates that there is still some room for improvement.

To sum up: the predictive skill of the novel spatio‐temporal censored GAMLSS model is competitive in comparison with station‐wise estimates, even for regions without observational sites.

The results show that the new spatio‐temporal censored GAMLSS model allows to accurate reproduction of the climatology over complex terrain, even for regions without observational sites.

## 5 Conclusion and discussion

A new method for estimating a spatio‐temporal precipitation climatology with a full‐distributional response and a daily temporal resolution is presented in this article. The climatology is represented by a GAMLSS using the new *R* package bamlss (Umlauf *et al*., 2016b) to optimize the regression coefficients. The estimated effects are shown in Section 4.1 and return interpretable and highly significant climatological features. An advantage of a full‐distributional model is that a variety of properties can be derived from the estimate. The novel climatology shows a good overall performance for the amount of precipitation on the daily scale, as well as for the probability of precipitation. The results demonstrate that the concept of censoring is suitable to account for the high number of zero observations in the data set. In contrast to the station‐wise reference methods shown in the article, the spatio‐temporal model returns fully distributional estimates for the whole area, even for regions where no observations are available. The cross‐validation shows that the spatial model returns accurate estimates for unobserved regions and is even able to out‐perform the station‐wise estimates in some cases.

The PIT histogram and the CP show that the model is overall well calibrated, but there is some room for improvement. Further adjustments of all tuning parameters (location *μ*, scale *σ*, but also the power parameter *p*) might have a positive effect on the results. Beside optimizing the parameters of the zero left‐censored normal distribution a different response distribution might bring additional benefits. Such distributions could be, e.g. a left‐censored logistic distribution (Messner *et al*., 2014), a gamma distribution (Rust *et al*., 2013; Wong *et al*., 2014), or a mixed distribution (Eden *et al*., 2014). Rust *et al.* (2013) has shown that a gamma distribution works well for precipitation on the original scale without the need to apply a power transformation, which might distort the data. On the other hand, the gamma distribution is not defined at zero. While Scheuerer and Hamill (2015) use a censored shifted gamma distribution, Rust *et al.* (2013) uses a two‐part approach where the probability of precipitation is modelled independently from the amount of precipitation. This allows use of the gamma distribution, but has the necessity to define and estimate two different models, while the approach presented in this article requires only the specification of one single model to obtain the full distribution and all its properties.

A direct comparison against more complex existing methods would be needed to explicitly highlight advantages and drawbacks of our method, but needs some extensions to our current model. Adding additional covariates beside the day of the year, longitude, latitude, and altitude could further improve the model results as shown in previous publications. Conceivable covariates could be, e.g. steepness and facing of the slopes, or the distance to the closest open water source. Furthermore, the new model allows inclusion of daily covariates, such as mean wind direction, covariates explaining the regional weather situation, and many others, which is not possible for longer aggregation periods (e.g. monthly). Some covariates have been tested but have not brought the expected results yet. The estimate of the statistical model (Equations (6)–(8)) can be performed in under 32 h on a single core (2.7 GHz Intel Xenon, 8 GB memory), although the model is already quite complex. However, estimating the full model including a ‘random’ set of covariates will be unsatisfying. One idea would be an automated iterative variable selection approach, such as boosting or ridge‐regression, to find the best additional covariates.

One big advantage of the method is that it is fully scalable as the model specifications are very general. While demonstrated for daily precipitation sums in this study, other aggregation periods would also be possible. The model estimation only requires the observations and corresponding covariates. To retrieve full‐probabilistic spatial estimates, a suitable digital elevation model (DEM) is needed. The resolution of the DEM predetermines the resolution at which the climatological estimates can be provided, wherefore the results can be served at a very high spatial resolution. As only few inputs are necessary (observations, DEM), and due to the very general framework, the method can easily be applied to other regions and data sets. Additionally, it would be worthwhile to apply the approach to other censored variables, such as wind speed, sunshine duration, or relative humidity, which would only require minor modifications.

## Derivation of the expectation function

Derivation of the expectation function for a power‐transformed zero left‐censored normal distribution as in Equation (5).

Assume a left‐censored normal distribution with a censoring point at zero without a power transformation. The distribution function *F*(*x*), and the density function *f*(*x*) are defined as follows:

(A1) $$F\left(x\right),\;f\left(x\right)=\frac{\partial F\left(x\right)}{\partial x}$$

and therefore the expectation of the distribution becomes:

(A2) $$E\left[x\right]=\int_{x=0}^{\infty }x\cdot f\;\left(x\right)\;dx$$

For a left‐censored normal power‐transformed distribution, the distribution function *G*(*z*) and density function *g*(*z*) can be written in the same way, where *x* from Equation (A1) is simply *z* ^1/*p*^:

(A3) $$g\left(z\right)=\frac{\partial G\left(z\right)}{\partial z}=\frac{\partial F\left(z^{1/p}\right)}{\partial z}$$

and therefore:

(A4) $$\frac{\partial F\left(z^{1/p}\right)}{\partial z}=f\left(z^{1/p}\right)\cdot \frac{z^{\left(\frac{1}{p}-1\right)}}{p}$$

leading to Equation (5) as shown in the article.
